# Induced Pluripotent Stem Cell Neuronal Models for the Study of Autophagy Pathways in Human Neurodegenerative Disease

**DOI:** 10.3390/cells6030024

**Published:** 2017-08-11

**Authors:** Natalia Jiménez-Moreno, Petros Stathakos, Maeve A. Caldwell, Jon D. Lane

**Affiliations:** 1 Cell Biology Laboratories, School of Biochemistry, University of Bristol, Bristol BS8 1TD, UK; natalia.jimenezmoreno@bristol.ac.uk (N.J.-M.); ps13590@bristol.ac.uk (P.S.); 2 Trinity College Institute for Neuroscience, Trinity College, Dublin 2, Ireland; maeve.caldwell@tcd.ie

**Keywords:** autophagy, mitophagy, autophagic flux, stem cells, pluripotency, hiPSC, neurons

## Abstract

Human induced pluripotent stem cells (hiPSCs) are invaluable tools for research into the causes of diverse human diseases, and have enormous potential in the emerging field of regenerative medicine. Our ability to reprogramme patient cells to become hiPSCs, and to subsequently direct their differentiation towards those classes of neurons that are vulnerable to stress, is revealing how genetic mutations cause changes at the molecular level that drive the complex pathogeneses of human neurodegenerative diseases. Autophagy dysregulation is considered to be a major contributor in neural decline during the onset and progression of many human neurodegenerative diseases, meaning that a better understanding of the control of non-selective and selective autophagy pathways (including mitophagy) in disease-affected classes of neurons is needed. To achieve this, it is essential that the methodologies commonly used to study autophagy regulation under basal and stressed conditions in standard cell-line models are accurately applied when using hiPSC-derived neuronal cultures. Here, we discuss the roles and control of autophagy in human stem cells, and how autophagy contributes to neural differentiation in vitro. We also describe how autophagy-monitoring tools can be applied to hiPSC-derived neurons for the study of human neurodegenerative disease in vitro.

## 1. General Introduction

The autophagy cytoplasmic quality control system supports the function and survival of diverse cell-types within most if not all tissues in the body. It is upregulated in response to environmental and cellular stresses, and consequently provides protection against most of the significant human diseases including cancer, neurodegeneration, cardiovascular disease, and diabetes. While work continues to map the cellular general signalling pathways that contribute to the initiation and coordination of autophagy responses to a variety of internal and external triggers, we are now entering an applied phase that requires an appreciation of autophagy regulation in those cells and tissues that are directed affected by disease. The expectation is that the basic control elements will be identical across different cells/tissues (e.g., the core autophagy machinery represented by the AuTophaGy-associated (ATG) family of proteins), but we are learning that specific cell-types possess distinct autophagy regulatory inputs at the transcriptional and post-translational levels. This raises an important question: how can we study the intricacies of autophagy control in defined, disease-linked human cell-types in vitro, while assuring the molecular accuracy of appropriate model systems? The application of human stem cell-derived model systems promises to provide the solution to this important challenge.

It is generally accepted that autophagy plays a vital protective role in brain. Targeted depletion of core autophagy genes in mouse models leads to progressive neuronal loss, often preceded by neuronal dystrophy, and often associated with the build up of toxic intra- and extra-cellular protein aggregates (reviewed in [1]). Tellingly, defects in autophagy pathways—including those caused by specific autophagy-specific patient mutations—have been strongly linked with neurodegenerative diseases in humans [1]; meanwhile, increasing autophagy activity in mouse models of neurodegenerative disease (e.g., Parkinson’s disease (PD)), can slow or prevent the progress of pathology (e.g., [2]). Thus, a general principle that autophagy is needed to protect long-lived, post-mitotic neurons from the rigours of sustained neuronal function in the brain has emerged. However, despite an enormous effort globally to understand the pathogenesis of neurodegenerative diseases, we are still uncertain of the exact causes of neuronal cell death in most cases, thus emphasising the need for faithful cell-based models to understand the molecular pathways involved (Table 1). One approach is to grow neurons in the laboratory from human stem cells that have the potential to become any cell-type. This technology has enormous potential for improving our understanding of cellular responses to disease-related stresses, and the impact of disease-associated genetic mutations on key cellular pathways [3], but it is essential that stem cell-derived cell-types are thoroughly characterised before assumptions can be made about how closely these mirror their counterparts in vivo. Equally, the in vivo cellular environment (including neighbouring cells and the surrounding extracellular matrix) should be taken into account in the experimental design, potentially necessitating complex 3D and co-culture-based approaches. In this review we will discuss the methodologies for autophagy research in human stem cell neuronal models. We also discuss how stem cell neuronal models are informing our knowledge of how patient genetic mutations impact on the autophagy pathway, and how this in turn contributes to the onset and progression of human neurodegenerative diseases. While this manuscript was in preparation, a review on a very similar topic was published by Jungverdorben et al. [4], so to avoid unnecessary overlap, we have focused more on the technical strengths and limitations of using stem technology in neuronal autophagy research.

## 2. Autophagy: Types and Regulation

Autophagy is a catabolic process in which cytoplasmic material is delivered to the lysosome for degradation and recycling. This provides the cell with the capability to rapidly remove toxic waste, and to repurpose non-essential material during times of stress. Autophagy can be non selective or highly selective depending on the induction pathways, and the autophagy machinery has adapted to sequester and degrade whole organelles (including mitochondria (mitophagy)), and invading microorganisms (xenophagy), which is does through the availability of adaptor proteins that recognise specific cargoes and recruit the autophagy machinery (e.g., P62/SQSTM1; optineurin; NDP52). Together, these properties make autophagy an essential component of cellular stress defence pathways, particularly in long-lived, post-mitotic cells that cannot dilute damaged/waste material via cell division. An obvious example of such a cell-type is the neuron. Here, autophagy contributes to axonal regeneration and presynaptic homeostasis [5,6]. It is needed to degrade neurotoxic factors (e.g., α-synuclein), as well as damaged organelles (particularly mitochondria) that are tagged by poly-ubiquitin and subsequently recognised by autophagy adaptors [7,8]. Consequently, its dysregulation has been linked to the development of neurodegenerative diseases [9], and crucially decreased autophagic activity is a characteristic of aging [10]. Suppression of basal (housekeeping) autophagy triggers neurodegeneration [11,12], and several ATG proteins have been linked to neuronal survival in the face of stress [13,14]. Clearly, a thorough understanding of autophagy regulation in neurons—particularly those affected in neurodegenerative disease—will be beneficial.

Autophagy can be divided into 3 distinct forms, each having an unique mechanism for lysosomal substrate delivery: microautophagy, in which material is delivered into the lysosome through a process of limiting membrane invagination; chaperone-mediated autophagy (CMA), which describes the selective lysosomal delivery of soluble protein cargo containing KFERQ-like motifs, via hsc70 and the LAMP2A receptor; and macroautophagy (described herein simply as “autophagy), which describes the de novo assembly of an isolation membrane that sequesters cargo within a ~500nm double membrane-bound autophagosome that ultimately fuses with the lysosome. It is not within the scope of this review to describe in detail the autophagosome assembly pathways, so the reader is instead directed to excellent, recent descriptions of this process [15,16,17], and we apologise to the many authors whose primary work we are unable to cite. In brief, the autophagy pathway is wired into the nutrient-sensing functions of the mTORC1 complex that monitors amino acid flux at the lysosome and responds to growth factor availability, and AMPK that detects changes in cellular ATP levels. These control autophagy via the ULK1 complex, which is assembled and activated during autophagy stimulus (e.g., starvation). The ULK1 complex consists of ULK1 (ATG1 in yeast), ATG13, ATG101, and FIP200 (FAK family kinase-interacting protein of 200 kDa; also known as RB1CC1 (RB1 inducible coiled-coil 1)). The active ULK1 complex is enriched at presumptive autophagosome assembly sites, where it engages downstream machinery including ATG9-positive vesicles and the autophagic class III phosphatidylinositol 3-kinase (PI3K). The PI3K complex contains Beclin1 (BECN1; ATG6 in yeast), VPS34 (the catalytic PIK3C3 subunit), VPS15, ATG14L (also known as Barkor), and NRBF2. When activated, the PI3K complex generates PI3P on the platform membrane (most typically of ER origin), and this in turn recruits PI3P effectors of the WIPI (WD repeat domain phosphoinositide-interacting protein 1) family, to engage downstream events.

At PI3P-enriched nascent assembly sites, autophagosome nucleation begins, and this process proceeds towards the autophagosome membrane expansion stage that requires the conjugation of ATG12 to ATG5, and of ATG8 to the lipid phosphatidyl ethanolamine (PE), mediated by E1- and E2-like proteins, ATG7, ATG3 and ATG10. ATG12-ATG5 associates with ATG16L1 to form a larger complex that is recruited to the isolation membrane in part via an interaction between ATG16L1 and WIPI2B [18]. This complex functions as an E3-like ligase to drive the local lipidation of ATG8 family members (including LC3A, LC3B, LC3C, GABARAP, GABARAP-L1, GABARAP-L2/GATE-16) that, in turn, coordinate autophagosome membrane expansion, cargo selection and membrane closure. Members of the ATG8 family of lipid modifiers are synthesised as immature precursors, and need to be activated by a cleavage event mediated by ATG4 endopeptidases, that reveals a C-terminal glycine residue for covalent attachment to PE. ATG4 also acts as an ATG8 deconjugase, releasing ATG8 into the cytoplasm for recycling by a mechanism that is coupled to the autophagosome maturation process. The sealed autophagosome then traffics to the lysosome, fusing en route with organelles of the endocytic network, by a process that is coordinated by various Rab GTPases, membrane tethers and SNARES [17].

## 3. Stem Cells in Laboratory Research: Focus on Autophagy

**Figure 1:**
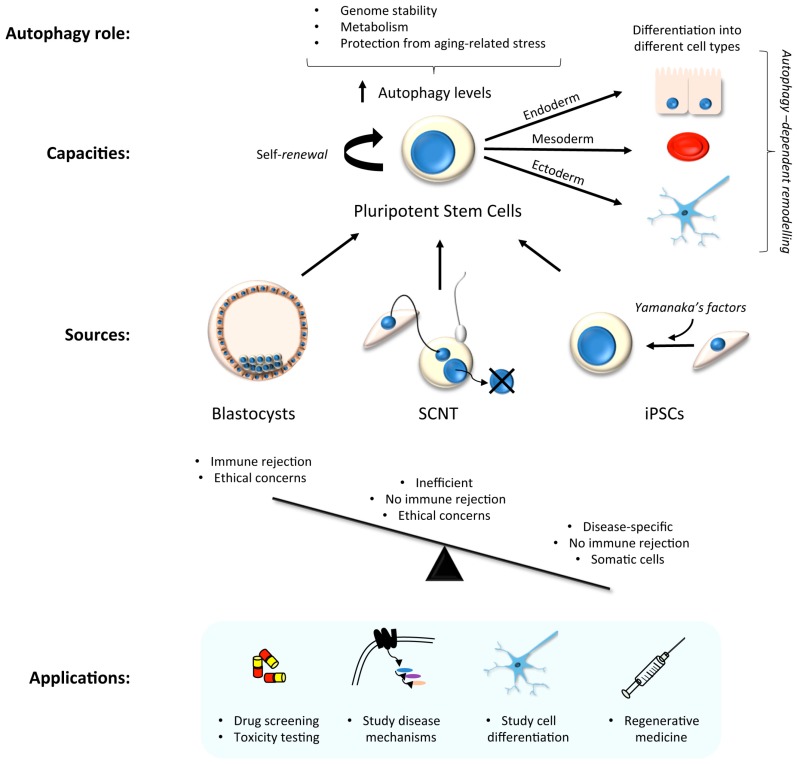
Schematic representation of different types of stem cells and their applications. Pluripotent stem cells can be obtained from human blastocysts, by somatic cell nuclear transfer (SCNT) into a recipient, enucleated oocyte, or by human induced pluripotent stem cell (hiPSC) reprogramming. Each has its advantages, with hiPSCs providing a source of cells for mechanistic research that also overcomes important ethical concerns. Autophagy levels have been reported to be high in pluripotent stem cell, and here autophagy coordinates metabolism, prevents genome instability, and protects against aging-associated stress. The requirement for autophagy during differentiation is tissue- and cell-type dependent.

Stem cells have two main capacities: self-renewal, and the ability to generate differentiated cell-types. There are two major classes of stem cells depending on their plasticity: embryonic stem (ES) cells which are pluripotent, hence can give rise to cells from each of the three embryonic germ layers, and thereby derive each of the myriad differentiated cell-types in the body; and somatic- adult stem cells, which are multipotent, hence can seed those lineage-specific cell-types that are found within a specific tissue or organ. Human ES cells have thus emerged as attractive tools for the development of cell and gene therapies [19]; however, their use is controversial [20]. Even without such barriers, there remains the problem of immune rejection in any cellular transplantation-based therapy, as ES cells are not genetically tailored to the patient. Alternative systems have been developed in order to bypass some of these problems. For example, ES cells can be generated by somatic cell nuclear transfer (SCNT) of patient somatic cell nuclear donation into the cytoplasm of enucleated human oocytes [21], although this does not entirely avoid ethical controversies (Figure 1).

Human induced pluripotent stem cell (hiPSC) technology has recently emerged as a powerful and exciting tool for both research and cellular transplantation [22,23,24,25]. This was first achieved by Yamanaka with the addition of factors that force cellular reprogramming towards a stem cell state [26], and this drives a cascade of molecular changes that promote and sustain “stemness” [27]. Reprogramming can be divided into 3 different phases: initiation, maturation and stabilization [28]. The initiation phase is stochastic, and is key to the loss of cell-type background and the continuation of reprogramming. The two last phases are deterministic, and are based on the activation of pluripotency genes, such as those of the Sox2 locus [29]. Once hiPSC lines have been generated, the timely addition of specific growth and patterning factors, cytokines, and inhibitors—in the correct order/combination/concentration—enables the generation of tissue-specific and also region-specific cell-types. This technology is therefore useful for the study of molecular disease mechanisms in pertinent cell-types, for drug screening again in the appropriate cell-type, and for the advancement of regenerative medicine [23,30,31] (Figure 1). HiPSC lines can be generated from patient fibroblasts and gene edited in vitro to provide isogenic controls and a source of differentiated cells to re-introduce into the patient for restorative therapy, thereby circumventing possible immune rejection concerns.

**Figure 2:**
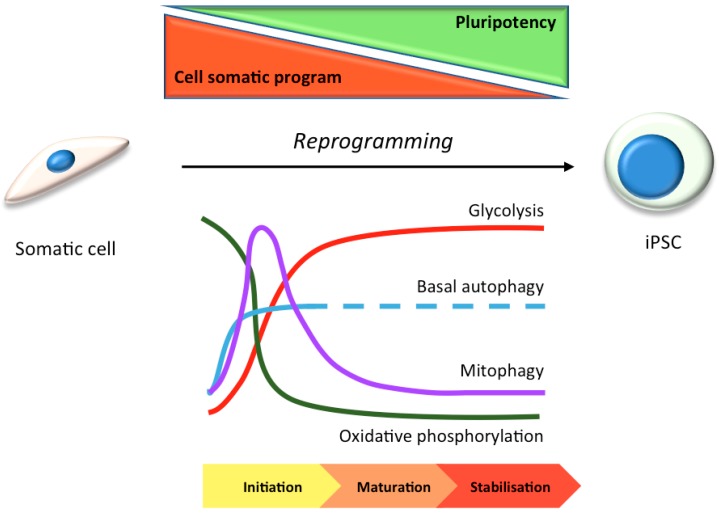
Schematic showing the control of autophagy and cellular metabolic status during the switch to pluripotency. Autophagy is upregulated during the initiation phase, and this correlates with complete mitochondrial depletion/renewal (via mitophagy and new biogenesis), and a metabolic switch from mitochondrial oxidative phosphorylation dependency, to glycolysis [45].

In many stem cell-types—including mesenchymal, epidermal, dermal, and hematopoietic stem cells—basal autophagy has been reported to be constitutively high [32,33]. This may be because stem cells are long-lived and require efficient homeostatic pathways to restrict the accumulation of protein aggregates and damaged organelles to remain healthy. High levels of autophagy in stem cells prevents genomic instability, maintains metabolism, and protects against aging-related factors like reactive oxygen species that can affect the self-renewal or differentiation capacity [34,35,36] (Figure 1). In hiPSC cells, the need to undergo an intense reshaping process—including a reduction in mitochondrial mass (see Section 3.1) [37,38]—correlates with elevated autophagic activity during the reprogramming phase [39,40] (Figure 2). Indeed, pharmacological induction of autophagy, through the inhibition of the mTOR or insulin pathways, improves hiPSC reprogramming efficiencies [41,42,43]; meanwhile, hyperactivation of the mTOR pathway inhibits somatic cell reprogramming [42]. Accordingly, reprogramming is inhibited by the loss of key autophagy proteins in mouse, such as Atg3, Atg5 and Atg7 [44]. It has been suggested that the four pluripotency factors used as standard to generate hiPSCs (OCT4, SOX2, c-MYC, KLF4) coordinately and transiently repress mTOR during the reprogramming phase, thereby upregulating autophagy (although the individual contributions of each factor during autophagy gene expression control are varied and complex [40,44]). The idea that autophagy activation is involved in the induction of pluripotency is somewhat countered by the suggestion that accumulation of P62/SQSTM1 (a selective autophagy substrate and adapter) is needed for effective reprogramming [40]. It is therefore argued that a subtle balance between the need for mitochondrial remodelling (mitophagy) during reprogramming, alongside a somewhat muted non-selective autophagy pathway is needed [40] (Figure 2).

### 3.1. hiPSC Neuronal Models and Autophagy

The ability to differentiate hiPSC into neurons has provided researchers with the tools to study human neuronal function and stress responses in vitro, and to carry out drug testing in applicable cell-line models. Until recently, the main resource for neuronal study in vitro had been primary neurons from rodent brains. This has obvious advantages in that cells can be obtained from specific brain regions (e.g., the hippocampus), and these quickly re-establish synaptic contacts, while the presence of support cells such as glia enables their maintenance in culture for up to ~8 weeks under the right conditions [46]. Research focused specifically on human neurons has mainly been restricted to post-mortem studies [47] or to studies using human cell-lines with neuronal characteristics (e.g., SH-SY5Y–a bone marrow-derived neuroblastoma cell-line that displays some properties of dopaminergic neurons, but can be driven towards other neuronal sub-types by the application of specific factors [48]). Primary human cultures (including human foetal cultures [49]) are limited by sample availability and by obvious ethical concerns. Biopsies of olfactory epithelium have been used for studies of oxidative stress in Alzheimer’s disease (AD) patient “neurons” (although these cells were not sufficiently characterised [50]), while tissue obtained from surgical procedures has been used for single cell transcriptomics, either directly [51], or following prolonged culture in vitro [52]. In recent years, a novel technique of direct reprogramming has been described in which fibroblasts are converted into neurons (termed iNeurons) by the addition of Ascl1, Brn2 and Myt1l transcription factors, thus skipping the de-differentiation (pluripotency) step [53]. This has great potential for studies in the laboratory and in the clinic, and although conversion rates typically remain quite low (~20% [54]), the neurons generated retain age-specific characteristics (unlike hiPSC-derived neurons), allowing the analysis of aging-related disease parameters (e.g., [55]). Interestingly, a similar approach can be used to directly convert astrocytes into dopaminergic neurons in vitro and in vivo [56], suggesting that this technique has the potential to allow the generation of specific types of neurons for research and/or regenerative medicine.

HiPSCs have the potential to differentiate into any of the distinct, region-specific classes of neurons and glia found in the brain. They capture the genetic diversity of the patient population, and are currently the most practical way to study human neural development in vitro, and to provide material for cell-based regenerative medicine [57,58]. Differentiation of hiPSCs to neural progenitors follows the same temporal course of neural differentiation of embryonic stem cells [59]. There are two main approaches to derive neural progenitors, and neurons thereafter: embryoid bodies (EB) [33,60,61] and adherent “monolayers” [62,63]. EB protocols are based on the formation of 3D dimensional cellular aggregates (e.g., neurospheres or organoids) comprising neurons alongside other cell-types, and being representative of a complex tissue. Monolayer-based protocols do not provide the same cell-to-cell connectivity, but have several advantages, including faster differentiation rates, a more homogeneous population of neurons, and sufficient dispersal to facilitate the study of individual neurons [64]. The choice of system will be determined by the type of analysis required (for a very useful comparison of culture systems, see [65]).

A specific combination of different factors guides cellular differentiation towards neural progenitors that have the capacity to generate specific neuronal subtypes. Thereafter, the differentiation of hiPSC-derived neural progenitors into terminally differentiated neurons is based on the recapitulation of the neuralisation process during neural development in vivo [66]. Neural differentiation is typically correlated with the expression of specific neural markers and the acquisition of electrophysiological activity [67], although hiPSC-derived neurons display electrophysiological characteristics indicative of a somewhat immature state [68]. Despite this, hiPSC-derived neurons can efficiently engraft into primate disease models [62,69], demonstrating that they are at least poised to integrate into existing, functional neuronal circuitry. Further limitations include the possible acquisition of genetic mutations (particularly affecting P53) during reprogramming [70], and “resetting” of ageing characteristics necessitating use of experimental interventions to study ageing parameters in hiPSC-derived neurons [71]. There are certain additional limitations in the use of hiPSC neuronal models, including: relatively long differentiation timescales; purity of neuronal populations in culture; the absence of defined protocols for the differentiation of certain neuronal sub-types; and the high costs of growth media reagents. Notwithstanding these considerations, there are numerous publications demonstrating the effective application of hiPSC neuronal models in a range of neuroscience-related topics [3,4,72,73]. In the following sections, we will explore how hiPSC neuronal models have been used to examine autophagy in important human neurodegenerative diseases, and the tools currently available.

## 4. Studying Autophagy in hiPSC-Derived Neurons

In the autophagy field, there are recognised standards for the monitoring of autophagic activity (including autophagic flux) that should be adopted in any cell-based assay [74]. Additional practical difficulties emerge when working with hiPSC-derived neurons—as with any neuron—but it is essential that the same rigour is applied if we are to be able to make predictions about autophagy regulation in disease. Herein, we will describe how autophagy monitoring methodologies can be used hiPSC-derived neurons, and by reviewing published examples of autophagy research in hiPSC-derived neuronal models, we will consider whether monitoring standards have been applied. One aspect that is often overlooked when assessing autophagy in neurons is their unique architecture, which means that events taking place in the cell body (soma) may not necessarily reflect the situation in the periphery and/or at local pre-and post-synaptic sites. As neuronal cell death in neurodegenerative disease is so often preceded by the loss of synapses and the collapse of neuronal processes, autophagosome assembly and retrograde transport kinetics in the periphery ought to be monitored in addition to events taking place in the soma. This poses a particular challenge in that it is often extremely difficult to confidently relate cell bodies with their associated extremities, and this approach is rarely used.

### 4.1. Activating Autophagy in hiPSC-Derived Neurons

Whilst in many cases it has been sufficient, and indeed often desirable to monitor autophagy activity in complete growth media, in the absence of stress—as is the case when comparing between control cells and those harbouring a disease-linked mutation, for example—the impact of a particular genetic mutation may not be apparent until the hiPSC neuronal culture is subjected to some form of autophagy-inducing stress. Difficulties with data interpretation arise when considering that different hiPSC lines can have very different growth properties in culture, meaning that their basal autophagy states may vary in a manner that does not necessarily reflect their differing genetic backgrounds. There are several ways in which this ambiguity can be avoided: (i) using several age-matched control and mutant background hiPSC lines; (ii) applying rigorous autophagy controls to confirm precisely where any differences in autophagy state originate (induction versus flux rates, for example); (iii) the use of isogenic controls to introduce disease-associated mutations, or to correct mutations in the same hiPSC background. For the latter, CRISPR/Cas-9 technology for gene editing is a very useful tool (see [75] for one relevant example), although the challenge of correcting/introducing complex genetic mutations, and issues with the requirement to isolate clonal hiPSC populations that might have subtly different properties and the possibility of off-target effects remain.

**Figure 3:**
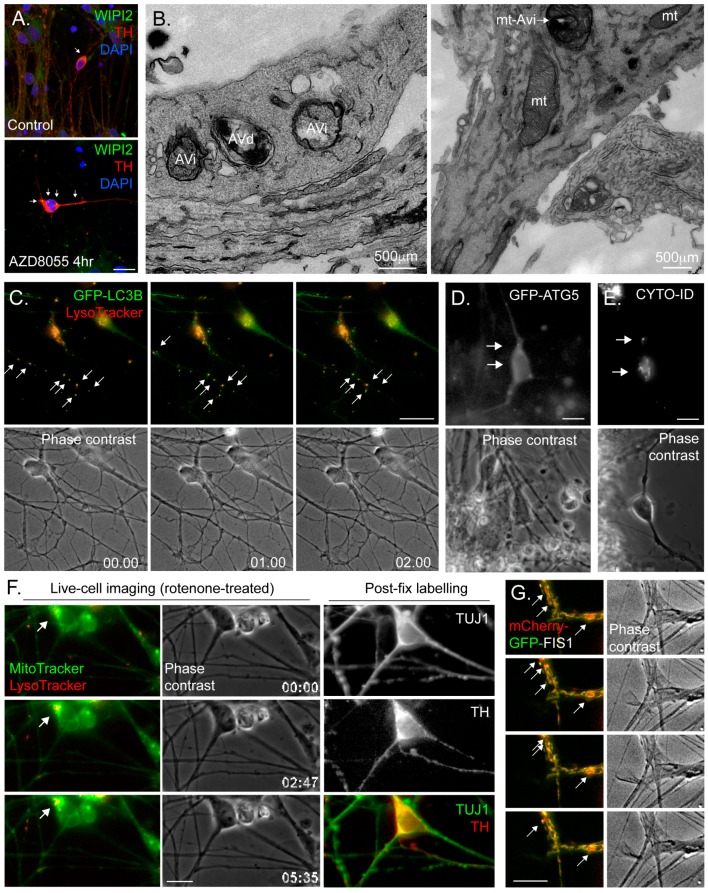
Example images of autophagy and mitophagy assays in dopaminergic (DA)-enriched human neuronal hiPSC cultures. (**A**) Fixed cell, confocal immunofluorescence imaging of DA neurons co-stained with anti-WIPI2 (autophagosome assembly site marker) and anti-TH (tyrosine hydroxylase; DA neuronal marker) antibodies. Note that the cell in the lower panel (treated with AZD8055) has a greater number of WIPI2-positive assembly sites, mainly restricted to the soma, indicative of upregulated autophagy. Scale bar = 20 µm. (**B**) TEM images of hiPSC-derived neurons in a DA neuron-enriched culture. Left: early autophagosomes (AVi) and autolysosomes (AVd) within a distended region of a neurite. Right: edge of a soma extending into a neurite, showing mitochondria (mt) and a mitophagosome (mt-AVi). (**C**) Live-cell imaging of hiPSC-derived neurons in a DA-enriched culture, expressing GFP-LC3B (introduced using a lentiviral vector) and co-stained with LysoTracker red (a fluorescent lysosomal stain). Arrows show dynamic autolysosomes (GFP-LC3B/LysoTracker-positive). Scale bar = 20 µm. (**D**) Live-cell imaging of a hiPSC-derived neuron in a DA-enriched culture, expressing GFP-ATG5 (an autophagosome assembly site marker, introduced using a lentiviral vector). Note the presence of 2 GFP-ATG5 positive assembly sites in the soma (arrows). Scale bar = 10 µm. (**E**) Live-cell imaging of a hiPSC-derived neuron in a DA-enriched culture, stained with the commercial autophagosomal dye, CYTO-ID. Note the presence of a cluster of autophagic structures in the soma, and an isolated autophagosome within a neurite (arrows). Scale bar = 10 µm. (**F**) Example of post-fix labelling for DA neurons in a hiPSC-derived neuronal culture. In this example, cells were co-loaded with MitoTracker green (fluorescent mitochondrial dye) and LysoTracker red, and imaged following treatment with the mitochondrial poison, rotenone, using a wide-field fluorescence microscope. Arrow depicts a neuron with strong MitoTracker/LysoTracker staining, which, following fixation and staining with anti-TH and anti-ßIII tubulin (TUJ1) antibodies was confirmed to be of DA status. Scale bar = 10 µm. (**G**) Wide-field, live-cell fluorescence microscopy demonstrating the use of the mCherry-GFP-FIS1 mitophagy reporter in neurons within a DA-enriched hiPSC culture (mCherry-GFP-FIS1 was introduced using a lentiviral vector). Arrows point to red-only mitolysosomes that can be quantitated as a measure of mitophagy on account of the quenching of GFP in the acidic lysosomal environment. Scale bar = 5 µm.

In more typical cell-line models, researchers normally test the autophagy response upon removal of nutrients and growth factors (starvation media). Following a brief lag (~15 min), a rapid induction phase is normally described, with autophagosome numbers reaching a plateau after around 30–40 min. During this phase, the synthesis and lysosomal degradation of autophagosomes are balanced (see below for discussion on autophagic flux). Starvation media is designed to inhibit mTOR (and therefore activate the autophagy pathway) through both amino acid depletion and growth factor removal, and is therefore extremely acute and wholly unphysiological. Indeed, treating hiPSC neuronal cultures with typical starvation media does not trigger a robust autophagy response in all neuronal sub-types (our unpublished observations); however, pharmacological mTOR inhibitors (e.g., rapamycin; AZD8055 (e.g., Figure 3A)) work well as do mTOR-independent autophagy inducers such as carbamezepine, verapin, and trehalose [76].

### 4.2. Taking Account of Autophagic Flux

Autophagic flux describes the entire process of autophagosome assembly, maturation, trafficking, merging with the lysosomal compartment (to form an autolysosome), and degradation in the lysosome. Any analysis of autophagosome numbers that does not take autophagic flux into account is flawed, simply because an apparent steady state change in autophagosome abundance cannot distinguish between altered rates of autophagy induction, or changes in lysosomal clearance efficiency [74]. The application of a lysosomal acidification inhibitor (e.g., Bafilomycin A1) or lysosomal protease inhibitors (e.g., leupeptin; E64D) is normally sufficient to allow assessment of autophagic flux: a further increase in autophagosomes in the presence of a lysosomal inhibitor signifies that autophagy is switched on; whereas similar autophagosome numbers in the presence of lysosomal inhibitors is consistent with a block in autophagosome maturation and/or lysosomal fusion. Additional considerations are needed when applying these parameters to neurons in culture, due to the spatial separation between peripherally-assembled autophagosomes and the lysosomal population, and the complex autophagosome assembly and transport-coupled maturation itinerary in these cells [77]. It is therefore important that researchers consider the different pools of autophagosomes found in neurons (those initiating in the soma versus those forming in the periphery, for example), as flux parameters are likely to differ between these.

The most often applied tool for directly measuring autophagic flux in non-neuronal model cell-lines is the “tandem-tag” (RFP-GFP or mCherry-GFP) LC3B construct [78,79], which relies upon the acidic nature of the lysosomal compartment and the differences in pKa values between the 2 fluorophores to compare early and late autophagic structures in fixed and living cells. Accordingly, early autophagosomes appear red/green under the microscope, whereas in autolysosomes with low pH, GFP is quenched resulting in the appearance of red-only punctate structures. Changes in the relative ratios of yellow (red/green):red puncta can therefore be used to assess autophagic flux in the absence or presence of lysosomal protease inhibitors. An interesting adaptation to this system was recently reported by Mizushima and colleagues [80]. Here, an expression construct is prepared consisting of GFP-LC3-mCherry-LC3 (G120A). This reporter works via the constitutive cleavage at the C-terminus of the first LC3 by the ATG4 endopeptidase to split the 2 distinct fluorescently tagged LC3 proteins: the GFP-tagged form can be lipidated and incorporated into nascent autophagosomes, ultimately reaching the lysosome where GFP is quenched; meanwhile the mCherry-tagged LC3(G120A) mutant cannot be lipidated and thus remains as an important expression reference in the cytoplasm. The green:red ratio therefore directly reports autophagic flux, and this reporter has been used in cell-lines, zebrafish and mice [80]. It is likely that the application of these flux reporters in hiPSC-derived neurons will become commonplace (e.g., [81]).

### 4.3. Common Autophagy Assessment Tools

Monitoring LC3B lipidation remains the front-line tool for rapid autophagy analysis in most autophagy research labs. This provides 2 readouts of autophagy induction: (i) the appearance of LC3B-decorated autophagosomal puncta in the cytoplasm of fixed or live cells; (ii) a gel shift in SDS-PAGE/immunoblotting assays (despite having greater mass than the unconjugated form, lipidated LC3 migrates faster on SDS-PAGE gels due to its increased hydrophobicity [82]). If careful consideration of autophagic flux is applied (Section 4.2), these assays are relatively fast and straight forward, but there are several other options that afford researchers with the opportunity to more accurately assess autophagy status, and the best advice is to use several approaches in tandem (see [74]). In this review series, Orhon & Reggiori describe in detail techniques for monitoring autophagy in cell culture [83]. In the following sections, autophagy monitoring tools will be outlined briefly with consideration given to the best way to apply these when using hiPSC neurons.

#### 4.3.1. Immunoblotting

The conversion of soluble LC3 (LC3-I) to the lipidated form (LC3-II) provides a direct readout for the amount of LC3 that is associated with autophagic membrane structures during macroautophagy—researchers can therefore correlate with some confidence the amount of LC3-II at a given time-point with the autophagic activity of the cell, provided that they account for autophagic flux. There are difficulties when applying this approach to hiPSC-derived neurons: (i) the amount of material required can limit the scope of any experiment; and (ii) hiPSC neuronal cultures can be quite heterogeneous, with other cell-types concurrent in the culture, meaning that such a population-based assay provides only a generalised picture of what is taking place in the dish. LC3B immunoblotting has been used to study autophagy in hiPSC-derived neurons in various conditions (e.g., [84,85]). Often, and in parallel, immunoblotting for P62/SQSTM1 and for components of the mTORC1/ULK1 signalling axis can be carried out to provide valuable supporting evidence for autophagic flux. Here, consideration must be given to the influence of de novo synthesis on the P62/SQSTM1 turnover rate (as is indeed also the case with LC3), particularly during prolonged autophagy induction.

#### 4.3.2. Immunofluorescence Microscopy

Fixing and labelling cells with antibodies for immunofluorescence microscopy is widely applied to cells and tissues for autophagy research. The detection of LC3 or P62/SQSTM1 puncta is a common way to monitor the number of autophagosomes and aggregated cargo, respectively, and this method has been used to study autophagy in hiPSC-derived neurons in different conditions [85]. Co-staining with antibodies against the lysosomal marker LAMP1, followed by co-localisation analysis to quantify autolysosomes [85], or measurements of LC3 puncta numbers in the absence or presence of lysosomal inhibitors provide readouts of autophagy flux. Researchers can also use antibodies that label the autophagosome assembly sites (e.g., anti-WIPI2 (Figure 3A)) to monitor changes in their abundance and/or location. The following should be taken into account when carrying out immunofluorescence studies with hiPSC-derived neurons: (i) in common with autophagy analysis in primary neurons, the architecture of this cell-type needs to be considered with respect to the location of autophagic structures and cargo—for example, fewer autophagosomes in the soma might indicate a disrupted autophagy pathway, but might equally reflect impaired retrograde autophagosome transport away from the periphery; (ii) fixation conditions can be more difficult to optimise when using neuronal cultures—for example, methanol fixation disrupts the integrity of delicate neurites, which appear “beaded” with bright staining, precluding analysis of distal punctate structures (e.g., Figure 3A); (iii) antibodies that work well in cultured epithelial and fibroblast cell-lines may not necessarily provide good staining in hiPSC neurons, so additional validation is needed; (iv) linked to the former, problems can be encountered when determining whether a punctate structure is a “true” autophagosome or, for example, immunoreactive or autofluorescent cell debris that tend to stick to neuronal processes; and (v) the need to counterstain for neuronal markers to ensure that the correct cells in the population are analysed restricts the availability of fluorescence channels and antibody options. We would recommend separate, quantitative analyses of autophagosome puncta in the soma and at peripheral sites (e.g., numbers of LC3B puncta per chosen length of neurite and/or within a given distance form neurite tips).

#### 4.3.3. Electron Microscopy (EM)

Transmission electron microscopy (TEM) remains a useful imaging standard to detect the presence of autophagic vacuoles in any cell-type [74]. Early autophagosomes are identified as double-membrane structures of ~500nm diameter, sometimes containing organelles, whose general content differs little from the surrounding cytoplasm (Figure 3B). More mature autolysosomes tend to present as single membrane-bound or multi-lamella vacuoles, often somewhat larger in size than earlier autophagic structures (Figure 3B). These can be distinguished from multivesicular bodies of the endocytic pathway by virtue of their more heterogeneous membranous content (for examples of TEM images of autophagic structures at different stages of maturity in primary human CD34+ stem cell-derived erythroid cells, see [86]). This method has been used to study autophagy in hiPSC-derived neurons in different conditions [85], and can be very useful for detecting changes in autophagic activity in disease-related hiPSC neurons. Caution needs to be applied when it is necessary to assess particular sub-classes of neurons in heterogeneous hiPSC-derived neuronal cultures, and immunoEM to detect suitable neuronal markers should be considered [87]. Here, it may be possible to use correlative light and electron microscopy (CLEM) using fluorescent reporters to identify certain types of cells.

#### 4.3.4. Fluorescence Live-Cell Imaging and Flow Cytometry

Vital dyes (e.g., MitoTracker; LysoTracker; redox reporters) work well in hiPSC neuronal cultures, and allow rapid and relatively straightforward live-cell imaging of organelle dynamics (Figure 3C,F) [88,89]. They are also applicable for flow cytometry analysis (e.g., [90]). GFP-based genetic reporters (e.g., GFP-LC3B (Figure 3C); GFP-ATG5 (Figure 3D); LAMP1-GFP; mito-dsRed; autophagy/mitophagy flux reporters (see Section 4.2)) are also available, and these are typically introduced into hiPSC cells or derived neuronal cultures via lentiviral vectors (e.g., [89]). Here it is important to identify the best promoter to drive expression in the neuronal cell-type of choice, since some commonly used mammalian promoters can be silenced in mature neuronal cultures (brain region-specific). Although there have been some outstanding imaging-based studies of autophagosome assembly and dynamics in primary mouse cultures—see for example the work of the Holzbaur lab using primary neurons obtained from the GFP-LC3B mouse [77,91], and the mitophagy work of the Schwarz lab [92]—these tools are not yet widely applied to hiPSC-derived neurons. In our lab, we are using the CYTO-ID reagent (Enzo Life Sciences)—a fluorescent dye that labels autophagic membranes with minimal cross over with lysosomes—for rapid autophagosome quantitation in hiPSC midbrain dopaminergic neuronal cultures (Figure 3E). Recently, a novel photoconvertable CMA reporter was described, allowing, for the first time, rapid assessment of CMA activity in living cells [93].

When using any live-cell imaging methodology with hiPSC neurons, the following should be considered: (i) neurons are relatively fragile cells which are less resistant to illumination-related oxidative damage, meaning that the duration of live imaging experiments and the exposure length/illumination intensity need to be taken into account; (ii) the highly asymmetric architecture of any neuron means that careful consideration of the location of any observed event is necessary (i.e., soma or neurite?); and (iii) the complexity of the neuronal culture—including the presence of additional, non-neuronal cell-types—means that it is often difficult to determine the relationships between cell body and neurite, and therefore direction of transport (retrograde or anterograde) of any dynamic organellar structures. For the latter, it is often desirable to image a population of neurons with relative few cells expressing a marker, so that these cells can be imaged in relative isolation (we refer the reader to the clear account of how to dilute cultures of GFP-LC3B expressing primary mouse neurons with a vast excess on non-expressing cells in Maday & Holzbaur [91]). Finally, when carrying out microscopy-based live-cell imaging experiments using hiPSC-derived neurons, it is essential that the precise identity of the cell being imaged is confirmed if we are to make accurate assumptions about autophagy control in a given neuronal sub-type. It is not unusual to encounter published examples in which the findings of imaging-based analyses have been assigned to rare neuronal sub-types within a hiPSC neuronal culture, without additional measures being applied to confirm neuronal identity (e.g., [89]). The approach that we take in PD-related work is to carry out live-cell imaging of hiPSC neuronal cultures that we have confirmed to be enriched with dopaminergic neurons (>50%), followed by fixing and labelling using anti-tyrosine hydroxylase (TH) antibodies (Figure 3F). Imaging parameters are then assessed only in those cells that have been retrospectively confirmed as TH-positive. This can be slow, painstaking work, but at least the data can be confidently attributed to the unique properties of those cells that are affected in disease.

#### 4.3.5. Monitoring Mitophagy

Given the strong correlation between mitochondrial dysfunction and neuronal decline and death in neurodegenerative disease, it is becoming increasingly important to monitor mitochondrial activity and quality control in human neuronal cultures. As mentioned above, there are vital dyes that measure mitochondrial parameters that can be applied to hiPSC-derived neuronal cultures (e.g., MitoTracker; MitoSOX). It is also possible to measure mitochondrial function in hiPSC neurons using, for example, Seahorse Analyzers (Agilent), but here it is important to take relative cell numbers and neuronal population characteristics into account. Fixed cell immunofluorescence analysis of, e.g., mitochondria/lysosome co-localisation and/or combined MitoTracker/LysoTracker live imaging can be used to assess mitophagy, provided that mitophagy flux is considered. Increasingly, researchers are using genetically-encoded mitophagy monitoring tools that act in a similar way to the tandem-tagged LC3 constructs (see Section 4.2) in that they emit different fluorescence signatures depending on whether mitochondria are located in the cytoplasm or in the lysosome. Mitophagy tools that have been developed and validated in vitro and in vivo include: mt-mKeima, a mitochondrial-targeted fluorophore (using the targeting domain of COX VIII) that shifts fluorescence characteristics from green to red when moving from neutral pH to acidic pH (such as in the lysosomal compartment) [94]; and tandem-tagged FIS1, a fusion construct consisting of the mitochondrial-targeted C-terminal tail of the outer mitochondrial membrane fusion factor FIS1 appended with GFP and mCherry; the former becoming quenched in the acid environment of the lysosome [95,96]. While either of these can in theory be used in hiPSC-derived neurons (we are currently trialling these in dopaminergic hiPSC neuronal cultures (e.g., Figure 3G)), the FIS1 construct has the advantage of being fixable.

#### 4.3.6. Gene Expression

Autophagy pathways are controlled at the transcriptional level by a number of key transcription factors, including TFEB and FOXO1/3 [97]. The availability of tools to monitor the actions of transcription factors can allow for rapid assessment of transcriptional pathways promoting autophagy. TFEB is a member of the MiTF/TFE family of basic helix-loop-helix leucine-zipper family of transcription factors. Under starvation conditions, TFEB stimulates lysosomal biogenesis and promotes autolysosomal efficiency by controlling the expression of more than 200 autophagy and lysosomal-related genes. It does so by binding to the CLEAR (Coordinated Lysosomal Expression and Regulation Network) elements of target genes. To monitor the induction the CLEAR gene network in cells, four tandem copies of the CLEAR sequence have been incorporated into luciferase expression constructs [98,99]. This assay has been applied successfully to iPSC-derived neurons [81,100], demonstrating that this general approach can be used to monitor transcription factor activity in these cells.

## 5. Autophagy Studies of Human Neurodegenerative Diseases Using hiPSC-Derived Neuronal Cultures

The idea that failures in autophagy quality control pathways contribute to the progress of neurodegenerative disease is based on the association of patient mutations in autophagy regulatory components, and on observations of accumulated autolysosomal structures in degenerating neurons. Using hiPSC technology, researchers are able to generate many (but not yet all) of the exact types of neurons that are lost in disease in order to study the regulation of autophagy in these cells in vitro, as well as being able to directly analyse the impact of patient mutations, either using hiPSCs derived from patients’ cells or using genome editing to generate isogenic hiPSC cell-lines. To this end, a number of hiPSC neuronal models have been generated that allow the study of autophagy and related neuronal stress response pathways in vitro, and the reader should consult [4] for a recent account of these. Given that autophagy provides protection against cell stress in long-lived cells such as neurons, it is perhaps unsurprising to find that that rare inherited genetic conditions associated with neurological defects have been identified that are caused by mutations in autophagy genes [101]. For these conditions, hiPSC technology is likely to provide a useful platform to pinpoint precisely where defects arise in the autophagy pathway. In the following sections, we will describe studies that have used hiPSC-derived neurons to study autophagy in common human neurodegenerative diseases, selecting examples that showcase the strengths and weaknesses of particular approaches.

### 5.1. Autophagy in hiPSC Models of Alzheimer’s Disease

Alzheimer’s disease (AD) is the most common neurodegenerative disease, and is characterized by the abnormal processing of the amyloid precursor protein (APP) through the amyloidogenic pathway, and the hyperphosphorylation of TAU protein. These two processes—along with an impaired activity of the proteolytic machinery, including autophagy—lead to extracellular accumulation of amyloid β (Aβ) peptides in senile plaques and aggregation of intracellular hyperphosphorylated TAU in neurofibrillary tangles (NFT) [102]. hiPSC-derived neurons have been used to study AD as they recapitulate some of the features characteristic of this disease, including an increase in Aβ secretion, reduced γ-secretase activity, and increases in phosphorylated TAU levels [24]. In this context, human iPSCs might be derived from either dominant familial AD (FAD) or sporadic AD (SAD) patients’ somatic cells [89]. The study of autophagy in iPSC-derived human AD neurons has improved our understanding of autophagy in this disease, and has facilitated the discovery of new factors, including: sphingomyelin metabolism; nuclear calcium signalling; and homocysteine exposure [81,100,103].

Lee et al. [81,100,103] studied autophagy dysfuntion in iPSC-derived neurons derived from FAD patient cells with presenilin-1 (PS-1) mutation following previous protocols [104]. In these neurons, acid sphingomyelinase (ASM) activity was increased, correlating with previous findings suggesting elevated ASM levels in the brain and plasma of AD patients. They analysed the presence of autophagic vacuoles using TEM, and found an increase in autophagic vacuole accumulation in PS-1 mutated neurons, and a decrease in TFEB target genes indicative of decreased autophagic flux. In addition, when they suppressed ASM, lysosomal biogenesis and autophagy activity were restored to normal levels [103]. Reddy et al. generated iPSC-derived human forebrain cortical neurons from AD patients with M146L and A246E mutations, as well as a PS-1 knockdown in control neurons [100]. They found a reduction in the CLEAR-luciferase reporter activity in iPSC-derived human AD neurons as a well as decrease in LC3II levels in PS-1 knockdown neurons, suggesting decreased autophagy initiation as well as flux potential. In addition, they found that PS-1 mutant neurons exhibited decreased nuclear calcium levels, and the restoration of these levels rescued mTORC1 tethering and initiation of the autophagy process [100]. The same group studied the potential role of the amino acid metabolite homocysteine (Hcy)—whose raised levels are considered to be a risk factor for AD—in the autophagy pathway and in the development of AD [81]. They used iPSC-derived forebrain cortical neurons [105], and found that exposure to Hcy caused reduced autophagic activity including: an increase in mTORC1 activity; a reduction in autophagy flux (assessed using tandem tag LC3); and a reduction in TFEB activity, suggested by the increase of the phosphorylated form of TFEB and inhibition of the CLEAR-luciferase reporter activity. In addition, increasing the concentration of Hcy in iPSC-derived cortical neurons resulted in an increase in A42/40 ratio, and these levels were restored when autophagy was pharmacologically induced using rapamycin or TAT-Beclin1 [81].

### 5.2. Autophagy in iPSC Models of TAUopathy

TAU is a microtubule-associated protein mainly expressed in mature neurons. In TAUophaties, like AD or frontotemporal dementia (FTD), TAU is abnormally hyperphosphorylated and is found in aggregated form within neurofibrillary tangles (NFT) [106]. hiPSC technology has been a useful tool to study the role of autophagy in TAUopathy. For example, Verheyen et al. studied the effect of the P301L TAU mutation [107]. They generated iPSC-derived cortical neurons [108], and these were transduced to express the mutated P310L form of TAU. They found an increase in TAU aggregation and hyperhosphorylation that was reduced after the induction of autophagy with rapamycin or trehalose, suggesting the important role of autophagy to remove TAU aggregates in human neurons [107]. Also, Silva et al. analysed the effect of the A152T TAU mutation from FTD patients on the function of the proteolytic machinery [109]. Neurons displayed aberrant TAU accumulation, and the authors found an upregulation of autophagy activity indicated by increases in key autophagy markers (LC3II, ATG12/5, P62/SQSTM1, LAMP1 and LAMP2A). In addition, the pharmacological induction of autophagy with rapamycin downregulated TAU expression and maintained cell viability [109].

### 5.3. Autophagy in hiPSC Models of Parkinson’s Disease

Parkinson’s disease (PD) is second only to AD in its prevalence, with >160,000 people projected to be afflicted with the disease in the UK alone by 2020. It is a progressive neurodegenerative disorder, which causes motor, physical and cognitive impairments, leading to major disabilities, with the signature presence of α-synuclein-rich Lewy bodies in the brain. PD is caused by the loss of dopaminergic (DA) neurons in the *substantia nigra* leading to the disruption of the nigrostatal pathway. Although PD is mostly idiopathic, 5–10% cases are familial in which specific mutations are associated with autosomal dominant and autosomal recessive forms of PD. To date, 17 genes with PD-causing mutations have been identified, and iPSC technology has been used to obtain hiPSC-derived dopaminergic neurons from many of these. The selective loss of midbrain DA neurons in patients with PD is due to their unusual physiology, in particular their exposure to elevated levels of oxidative and energy stress [110]. It is therefore essential that researchers very carefully characterise iPSC-derived DA neuronal populations before making assumptions about autophagy control within these cells. Methods to generate midbrain DA neurons from hiPSCs include lentiviral-mediated overexpression of the midbrain transcription factor, LMX1A in combination with patterning factors [111], and by the timed application of inhibitors and patterning factors alone [62]; however, these do not generate pure midbrain DA neuronal populations (e.g., double FOXA2/TH-positive), meaning that markers should be used as standard when interpreting data. The application of hiPSC technologies to study neuronal processes including autophagy control in PD has been covered extensive elsewhere [4,65], so we will focus on a few prominent studies as exemplars of the use of autophagy assessment tools in hiPSC-derived neurons.

Autophagy flux has been studied in iPSC-derived DA neurons from PD patients (idiopathic or having the familial G2019S mutation in Leucine-Rich Repeat Kinase 2 (LRRK2)), using the LMX1A overexpression protocol [85]. After 75 days in culture, DA neurons from the PD patients possessed fewer and shorter neurites than aged/gender-matched healthy controls, and in some cases neurites were entirely absent—a phenomenon that has been previously associated with impaired autophagy [112]. Correspondingly, significantly increased numbers of autophagosomes and raised levels of P62/SQSTM1 were recorded in untreated idiopathic and LRRK2 derived DA neurons, indicative of defective basal autophagic flux (confirmed using lysosomal inhibitors in the absence/presence of rapamycin) [85]. To support these findings, TEM was used to show that the numbers of autophagosomes in PD-derived neurons were significantly higher than in controls. This approach also revealed accumulation of lipid droplets and the presence of dilated ER in the PD lines [85]. The hiPSC LMX1A overexpression differentiation protocol has also been utilised to investigate CMA regulation in PD. For instance, Orenstein et al. detected compromised lysosomal degradation of LRRK2 via CMA in dopaminergic neurons derived from PD patients carrying the G2019S-LRRK2 mutation due to abnormal accumulation of α-synuclein [113]. They found that α-synuclein levels were significantly higher and showed greater co-localisation with LAMP2A in PD lines in comparison with their age/gender matched controls, after 30 days of differentiation [113]. Furthermore, knocking down LAMP2A further increased α-synuclein levels and suggested that the G2019S-LRRK2 mutation attenuates α-synuclein clearance via CMA [113].

The most common hiPSC DA neuronal differentiation protocols mimic the midbrain DA neuronal specification pathways in vivo [66]. Protocols apply small molecules—the SB431542 (activin/nodal) inhibitor, and LDN193189 (BMP inhibitor)—that induce neuronal fate by blocking the TGFβ pathway via inhibition of the SMAD2/3 and SMAD1/5/8 (dual SMAD inhibition) signalling cascades, respectively. Midbrain DA identity is acquired by application of the patterning factors SHH, FGF8a and CHIR99021 (GSK3B inhibitor; WNT agonist) in the presence of knockout serum replacement (KOSR) medium which is progressively exchanged for N2 and B27 supplemented medium. The neural inducers and patterning factors are eventually replaced with BDNF (brain-derived neurotrophic factor), ascorbic acid, GDNF (glial cell line-derived neurotrophic factor), TGF3, dibutyryl cAMP, and DAPT for terminal differentiation and maturation [62]. Schondorf et al. used this protocol to generate DA neurons from iPSCs obtained from PD patients with acid α-glucocerebrosidase (GBA1) mutation (15-20% TH +ve), in order to demonstrate the association of this mutation with dysfunctions in the autophagic/lysosomal system [114] (see Section 5.6 for more detail on GBA1 and Gaucher’s disease). Co-staining for lysosomal LAMP1 and the autophagosomal LC3 markers revealed significantly increased numbers of lysosomes and autophagosomes in the PD-derived neurons in comparison with controls in basal conditions. On the other hand, treatment with the lysosomal degradation inhibitors leupeptin and ammonium chloride (NH_4_Cl) caused significantly lower levels of autophagosomal-lysosomal co-localisation (autolysosomes), suggesting impaired autophagosome content degradation as would be expected with mutations associated with lysosomal storage diseases. Using a modified Studer protocol [62,105], Fernandes et al. generated midbrain DA neurons using iPSC from PD patients with the GBA-N370S mutation [87]. They recorded increased autophagosome numbers associated with elevated beclin1 and P62/SQSTM1 levels in the GBA-N370S lines. Additionally, LAMP1, LAMP2 and cathepsin D expression was higher, and careful TEM analysis (using anti-TH immunolabelling) revealed distended lysosomes with accumulated cargo in DA neurons, demonstrating impaired lysosomal degradation and therefore, deficient autophagic flux [87].

Two research groups have used the Studer protocol [62,105] to study mitochondrial properties and quality control in midbrain DA neurons derived from patient-derived iPSCs with mutations in the E3 ubiquitin ligase, Parkin or in PINK1 (PTEN-induced putative kinase 1) [115,116]. Valinomycin-induced mitochondrial Parkin recruitment was attenuated in PINK1 mutant DA neurons, and this was rescued by overexpression of WT-PINK1 [115]. Based on the recorded elevated mtDNA levels in mutated PINK1 lines, these authors hypothesised that mitophagy was deficient, although increased levels of PGC1α suggested a parallel compensatory mechanism to restore mitochondrial dysfunction [115]. In Parkin and PINK1 mutant iPSC-derived DA neurons, enlarged mitochondria and elevated mitochondrial oxidative stress have been recorded, suggestive of defective mitophagy quality control [115,116]. Using a similar differentiation system, Suzuki et al. used FACS-sorting with CD184^high^/CD44^−^ markers, in an effort to purify DA neurons from iPSCs from patients with Parkin mutations [117]. They reported impaired mitophagy in PD lines in comparison with the controls using the mt-mKeima reporter (see Section 4.3.5) [117].

Using a patterning protocol that is based on doses of FGF8a, WNT1 and retinoic acid [118], Hsieh et al. studied mitochondrial dynamics and mitophagy in iPSC-derived DA neurons from patients with the LRRK2-G2019S mutation [89]. They described differences in stress-associated mitochondrial motility parameters, and recorded a slowing of Miro degradation during mitochondrial stress in LRRK2-G2019S mutant iPSC-derived neurons. This was associated with delayed optineurin and GFP-LC3 recruitment to damaged mitochondria [89]. To examine mitophagy, they used mt-mKeima, again finding a delay in mitochondrial quality control in LRRK2-G2019S. Overall, these extensive studies clearly show how one particular PD-associated mutation affects mitochondrial behaviour and quality control, although with such a low proportion of DA neurons in the cultures (10-12%), it remains to be determined that these parameters accurately describe the situation in PD-vulnerable neurons. Finally, autophagy induction has been studied in neurons harbouring the PD-associated R258Q synaptojanin 1 mutation (in the SAC1 domain) [119]. Synaptojanin 1 is a lipid phosphatase which drives synaptic endocytosis, and the R258Q mutation alters PtdInsP signalling. Vanhauwaert et al. used iPSC-derived neurons from 2 PD patients with the R258Q mutation (and 2 age-matched controls) to study role of synaptojanin in neuronal autophagosome formation (note: these were not midbrain DA neurons) [119]. They found an increase in WIPI2 puncta in neurons harbouring the mutation, and this was emphasised in starvation conditions, suggestive of defects in the translation of the PI3P signal into productive autophagosome assembly [119].

### 5.4. Autophagy in hiPSC Models of FTD

FTD is a neurodegenerative disorder with brain atrophy involving the frontal and anterior temporal lobes [120]. It is characterised by abnormal accumulation of TAU protein (see Section 5.2) and TAR DNA-binding protein 43 (TDP43) [121]. FTD can also be present in motor neuron diseases, including amyotrophic lateral sclerosis (ALS). ALS is characterised by the loss of cortical and spinal motor neurons at the spinal or bulbar level. Whilst the cause of ALS remains undetermined in many patients, it is often linked to mutations in superoxide dismutase 1 (SOD1). Other genes known to cause ALS associated with FTD include the hexanucleotide repeat expansion in C9ORF72 gene, and mutations in progranulin or in TDP43. Almeida et al. generated iPSC-derived neurons from FTD-ALS patients with the GGGGCC repeat expansion in the C9ORF72 gene [122]. They found that treatment with chloroquine or 3-methyladenine (3-MA—inhibits autophagy via PI3K) decrease cell viability in FTD-ALS neurons. In addition, they found an increase in P62/SQSTM1 levels by immunoblotting, suggesting that autophagy function might be compromised (although LC3-II levels were not assessed) [122]. Meanwhile, Holler et al. studied progranulin (PGRN) haploinsufficiency through the generation of iPSC-derived neurons from FTD patients with GRN mutations [123]. They identified trehalose as a novel autophagy modulator of PGRN levels in FTD neurons. To characterise the effect of a pathogenic TDP43 gene (TARDBP) mutation, Baramada et al. generated iPSC-derived motor neurons from ALS patients [124] as previously described [125]. They proposed that two potent autophagy inducers, fluphenazine and methotrimeprazine, can enhance cell survival in TARDBP neurons, suggesting the induction of autophagy as a good strategy for ALS/FTD. However, no further characterisation of the autophagy process was carried out.

### 5.5. Autophagy in hiPSC Models of Other Neurodegenerative Diseases

Huntington’s disease (HD) is caused by a mutation in the gene encoding huntingtin (htt). It is characterised by the loss of medium spiny neurons. Previous studies in mouse and using human tissue have proposed that alterations in autophagy contributes to the development of HD and the accumulation of htt suggests a dysregulation of the autophagy pathway [126,127]. Nekrasov et al. generated GABAergic-like neurons from HD patients [90]. They showed a recapitulation of some of the HD features including huntingtin protein aggregation and an increase number of autophagosomes/lysosomes using flow cytometry and electron microscopy. In addition, normal levels were restored with a drug previously identify in mouse as a possible treatment for HD [128].

Charcot-Marie-Tooth 2A (CMT2A) is a sensorimotor neurodegenerative disorder most often caused by mutations in the mitochondrial fusion protein, MFN2 [129]. To analyse the involvement of autophagy quality control in disease progress, Rizzo et al. generated spinal motor neurons from hiPSC derived from CMT2A patients [130]. They reported an induction of general autophagy genes such as TFEB and ATG5, and most importantly, several genes involved in mitophagy control including PINK1, PARK2 (Parkin), BNIP3 and Beclin1. They also found an increase in LC3II levels and a reduction of P62/SQSTM1 by immunoblotting, and increased colocalization of mitochondria and lysosomes in the mutant cells, supporting an increase of autophagy levels, particularly mitophagy, in CMTA2 motor neurons [130]. Whether this increased mitochondria/lysosomal colocalisation was due to decreased mitophagy flux was not measured.

Spinocerebellar ataxia type 3 (SCA3) is the most common dominantly inherited form of cerebellar ataxia. It is characterised by the misfolding of the ataxin 3 (ATXN3) protein due a CAG trinucleotide expansion in the *ATXN3* gene. To analyse the effect of this mutation in neuronal autophagy, Ou et al. generated hiPSC-derived neurons from SCA3 patients using a commercial neural-induction media that specifies a general neuronal population [131]. They found an increase in the turnover of mutated ATXN3 upon autophagy induction using rapamycin in comparison to the wild type ATXN3 [131], an interesting observation given the recent demonstration that ATXN3 interacts with and controls the stability of beclin-1 to regulate autophagy [132].

Tuberous sclerosis complex (TSC) is a neurodevelopmental disease characterised by the formation of hamartoma in almost every organ. It is caused by mutations in the genes TSC1 or TSC2, required for the activation of the mTORC1 kinase. Ebrahimi-Fakhari et al studied the effect of TSC1 and TSC2 mutations in human neurons particularly in mitochondrial function [133]. They generated iPSC-derived cortical neurons from TSC patients (using a neurogenin-2 overexpression protocol [134]), and found an increase in the mitochondrial population with low TMRE signal (fluorescent dye that accumulates in polarised, active mitochondria), suggesting an accumulation of depolarized mitochondria [133]. This could indicate dysfunctions in the mitophagy process, however this was not formally assessed.

### 5.6. Autophagy in hiPSC Models of Neuronal Lysosomal Storage Diseases

Gaucher disease (GD) is a lysosomal storage disorder caused by mutations in the acid glucosylceramidase (GlcCerase; GBA1) gene, leading to the accumulation of glucosylceramide in the cell. The severe forms of GD include a low (Type 2) or fast (Type 3) progressive neurodegeneration [135]. Awad et al. used iPSC-derived generic neurons from GD patient iPSCs to study lysosomal alterations and the pathological changes affecting neurons with GBA1 mutation [136]. They found a reduction in lysosomal number—with a decrease in LAMP1 and LAMP2 levels—and a block in autophagic flux due to defective lysosomal clearance of autophagosomes in GD neurons (LC3II levels were not altered after chloroquine treatment, and the colocalization of LC3-GFP with LAMP1 was reduced with GBA1 mutations) [136]. In addition, reduced lysosomal biogenesis due a downregulation in TFEB expression was recorded in GD neurons, whilst GCase treatment restored lysosomal number and autophagy levels [136].

Niemann Pick type C1 (NPC1) is a rare lysosomal storage disorder leading to severe neurodegeneration and liver failure. It is caused by mutations in the cholesterol transporter NPC1, present in the lysosomal membrane. Previous work in hES-derived neurons suggested that aberrant autophagy control is associated with NPC1 neurons [137]. In this context, Maeztel et al. generated iPSC-derived neurons and hepatocytes derived from NPC1 patient iPSCs, with an isogenic control line prepared using TALEN technology [76]. In NPC1-derived neurons, they found cholesterol accumulation, an increase in the number of autophagic vacuoles (assessed using EM), and an increase in LC3II and P62/SQSTM1 levels by immunoblotting [76]. Notably, BafA1 treatment did not increase LC3II levels in NPC1 neurons, suggesting a block in autophagic flux [76]. Importantly, pharmacological induction of autophagy using rapamycin, carbamezepine, verapin and trehalose treatments reduced P62/SQSTM1 levels in NPC1 neurons [76]. By contrast, Lee et al. studied the effect of the vascular endothelial growth factor (VEGF)/sphingosine kinase pathway, and showed activity is reduced in NPC1 patients [138]. They generated iPSC-derived NPC1 neurons from NPC1 patients, and found, as previously described, an increase in LC3II and P62/SQSTM1 and a block in autophagy flux as determined by TEM and using mCherry-GFP-LC3B [138]. These levels were reduced after the treatment with VEGF [138]. Following this path, Soga et al. [139] studied the effects of cholesterol accumulation in iPSC-derived neurons from NPC1 patients, and found an increase in in LC3II and P62/SQSTM1 levels by immunoblotting that could be restored when cholesterol levels were reduced using 2-hydroxypropyl-γ-cyclodextrin and 2-hydroxypropyl-β-cyclodextrin (2HPβCD) [139]. Finally, Dai et al. used a variety of flux assays to show that methyl-β-cyclodextrin, an analogue of 2HPβCD, can restore autophagy levels in iPSC-derived neurons from NPC1 patients through the activation of the AMPK pathway [140].

### 5.7. Autophagy in hiPSC Models of Ocular Diseases

hiPSC have been differentiated into retinal ganglion-like neurons following affinity purification using the retinal marker THY1 to study the pathogenesis of glaucoma—an eye disease characterised by the loss of retinal ganglion cells in the optic nerve [141]. hiPSC-derived retinal cells from normal tension glaucoma patients with duplications in the TBK1 gene were reported to have an increase in LC3II levels [141]. Retinitis pigmentosa is a disorder that is characterised by the loss of the light-sensing photoreceptor cells of the outer neural retina. Retinal organoids established in 3D culture using Retinitis Pigmentosa patient-derived hiPSCs with mutations in TRNT1 (tRNA nucleotidyl transferase 1) showed altered autophagy activity, including an increase in LC3II levels and in elevated oxidative stress [142]. Further work will be needed to determine whether these changes in LC3 lipidation are caused by upregulated autophagic activity or impaired flux.

## 6. Future Perspectives and Challenges

Over recent years, progress in the application of iPSC neural differentiation protocols has provided researchers with an unrivalled opportunity to study the cell biology of human neurodegenerative diseases in vitro. In particular, iPSC-derived human neuronal cultures are enabling careful dissection of the roles of essential cellular protective pathways—such as the autophagy/mitophagy cytoplasmic quality control systems—in neuronal stress responses. Importantly, by carefully selecting the correct differentiation conditions, it is now possible to generate neurons in culture that faithfully preserve brain region-specific properties—an important consideration when studying the biology of vulnerable neuronal sub-classes that are often present in relatively small numbers in the brain. That said, we have somewhat rushed to find explanations for genetic mutations using patient iPSC-derived neurons, often without fully understanding the core biology of “wild-type” state, and studies continue to use population-based assessments of generic or heterogeneous neuronal cultures to make assumptions about the properties of rare, disease-affected neuronal sub-types. Challenges therefore remain in achieving accurate neuronal classifications and characterisation in iPSC neuronal cultures, and with developing complex 3D organotypic culture systems for accurate neuronal functionality tests in tissue models. There are clearly also some challenges when applying common autophagy tools to iPSC-derived neurons in order to accurately measure those autophagy parameters that we take for granted when using undifferentiated laboratory cell-line models [74,83]. As we move ahead, it is likely that more of these will be applied as standard in autophagy studies using iPSC-derived neurons, and through their careful use, we will inevitably learn more of how autophagy pathways contribute to neuronal function and survival in complex human neurodegenerative diseases, and how these can be exploited for neuroprotective and/or neurorestorative therapies.

## Figures and Tables

**Table 1:** Advantages and disadvantages of various assays systems to monitor human neuronal function in vitro. Green boxes indicate positive properties; pink boxes, negative properties.

Postmortem studies	Stable cell lines	Primary human cultures	Biopsies	iNeurons	iPSC-derived neurons
Brain connectivity	Soma neuronal characteristics	Non-tumour derived	Non-tumour derived	Direct reprogramming	Somatic cells
Disease-specific	Unlimited supply	Recapitulation in vivo neurons	Disease-specific	One-step process	Recapitulation in vivo neurons
Sample limitation	Physiological differences	Ethical concerns	Sample limitation	Disease-specific	Large supply
Static view	No disease-specific	Sample limitation	Surgical procedures	Regenerative medicine	Disease-specific
				Age-specific characteristics	Regenerative medicine
				Inability to expand	Study neurogenesis
				Low efficiency	Immature characteristics
					Variable efficiency (higher than iNeurons)
